# Assessing the Impact of Novel *BRCA1* Exon 11 Variants on Pre-mRNA Splicing

**DOI:** 10.3390/cells13100824

**Published:** 2024-05-11

**Authors:** Halla Elshwekh, Inas M. Alhudiri, Adam Elzagheid, Nabil Enattah, Yasmine Abbassi, Lubna Abou Assali, Ilenia Marino, Cristiana Stuani, Emanuele Buratti, Maurizio Romano

**Affiliations:** 1 International Centre for Genetic Engineering and Biotechnology, Padriciano 99, 34149 Trieste, Italy; halla.shwekh@icgeb.org (H.E.); yasmine.abbassi@icgeb.org (Y.A.); lubna.abou@icgeb.org (L.A.A.); ilenia.marino@icgeb.org (I.M.); stuani@icgeb.org (C.S.); 2 Department of Genetic Engineering, Libyan Biotechnology Research Center, Tripoli P.O. Box 30313, Libya; inas.alhudiri@btc.org.ly (I.M.A.); elzagheid@btc.org.ly (A.E.); nabil.enattah@yahoo.com (N.E.); 3 Faculty of Medicine, University of Southampton, Southampton SO17 1BJ, UK; 4 Department of Life Sciences, University of Trieste, Via A. Valerio, 28, 34127 Trieste, Italy

**Keywords:** *BRCA1*, exon 11, breast cancer, germline mutations, splicing, loss of heterozygosity, c.1019T>C, c.2363T>G, c.3192T>C, V340A, V788G, S1064S, functional genetics

## Abstract

Our study focused on assessing the effects of three newly identified BRCA1 exon 11 variants (c.1019T>C, c.2363T>G, and c.3192T>C) on breast cancer susceptibility. Using computational predictions and experimental splicing assays, we evaluated their potential as pathogenic mutations. Our in silico analyses suggested that the c.2363T>G and c.3192T>C variants could impact both splicing and protein function, resulting in the V340A and V788G mutations, respectively. We further examined their splicing effects using minigene assays in MCF7 and SKBR3 breast cancer cell lines. Interestingly, we found that the c.2363T>G variant significantly altered splicing patterns in MCF7 cells but not in SKBR3 cells. This finding suggests a potential influence of cellular context on the variant’s effects. While attempts to correlate in silico predictions with RNA binding factors were inconclusive, this observation underscores the complexity of splicing regulation. Splicing is governed by various factors, including cellular contexts and protein interactions, making it challenging to predict outcomes accurately. Further research is needed to fully understand the functional consequences of the c.2363T>G variant in breast cancer pathogenesis. Integrating computational predictions with experimental data will provide valuable insights into the role of alternative splicing regulation in different breast cancer types and stages.

## 1. Introduction

Pathogenic variants in the *BRCA1* gene greatly increase the risk of developing breast and ovarian cancer at a young age. 

*BRCA1* variants have been found distributed across the entire gene, with over 35,000 cataloged in the BRCA Exchange database as of 18 December 2023 (https://brcaexchange.org). However, determining the clinical impact of each variant is complex. The ClinVar database [1] (https://www.ncbi.nlm.nih.gov/clinvar/, as of 18 December 2023) contained classifications for over 3850 *BRCA1* variants as pathogenic and 388 as likely pathogenic on this date. Despite all this work, nearly 2700 variants still had conflicting interpretations of pathogenicity. Additionally, over 1750 variants were categorized as having uncertain clinical significance, meaning more evidence is needed to classify their disease risk. As a result, the clinical understanding of many *BRCA1* variants remains limited or inconsistent.

To enhance our understanding of these variants and provide valuable evidence for clinical care, functional studies are therefore essential. A significant proportion of *BRCA1* variants have been shown to disrupt splicing, especially within exon 11 because of its substantial size and functional significance. As alternative splicing plays a major role in cancer susceptibility, characterizing exon 11 variants of uncertain significance (VUS) is critical. Exon 11 (formally known as exon 10, transcript ID: ENST00000357654) encodes key domains and motifs, including Nuclear Localization Signals (NLS), interaction regions with crucial transcription factors, and DNA repair proteins [2,3]. It encodes key protein domains and interaction sites involved in nuclear localization, transcription, and DNA repair. Different splicing variants in this exon can thus considerably influence *BRCA1* function and include the Full-Length (FL) isoform, Δ11, and Δ11q [4]. The Δ11 isoform arises from alternative splicing events that exclude the whole Exon 11, resulting in a protein with modified functions compared to the FL isoform, potentially affecting DNA repair and cancer susceptibility. On the other hand, the Δ11q isoform is the result of the use of an alternative donor splice site within Exon 11, causing the exclusion of 3309 nucleotides that can potentially disrupt cell cycle regulation, DNA repair mechanisms, genome stability, and can also influence therapeutic response [5]. Additionally, the lack of NLS in this isoform may affect its cellular localization and function.

Characterizing the impact of novel VUS within *BRCA1* Exon 11 mutations is thus critical for better understanding the genetic predisposition to breast and ovarian cancers of individuals. In African countries, breast cancer is the second most common cancer among women over the age of 50 [6] and it accounts for over 25% of cancer cases in western Africa [7,8,9]. Challenges in organizing preventive screenings and accessing medical care contribute to delayed diagnoses and high mortality rates [10,11]. Additionally, the still understudied evolving genetics of African breast cancer further complicate the situation [12,13,14,15].

As a result, *BRCA1* mutations’ specific impact and prevalence in Africa are still very poorly understood. Therefore, comprehensive screening initiatives in this region are urgently needed. In this study, we utilized a minigene system to investigate the effects of three novel synonymous *BRCA1* mutations found in Libyan patients on splicing regulation. Our findings demonstrate that at least one of these variants alters exon 11 splicing, providing insights into its potential contribution to breast cancer predisposition. By dissecting the functional impact of *BRCA1* VUS on splicing, our work contributes to the interpretation of variants relevant to cancer risk and prevention strategies and expands the knowledge of *BRCA1* genetics in northern Africa.

## 2. Materials and Methods

### 2.1. Novel Variants of Uncertain Significance

The three novel VUS characterized in this study (Table 1) were identified during a genetic screening carried out in the Oncology Departments of the main public hospitals in Tripoli (Tripoli Central Hospital and Tripoli Medical Center). Complete clinical information on the analyzed patients is reported in a MedRxiv preprint [16].

The *BRCA1* (Accession: NM_007294.4) sequence from the National Center for Biotechnology Information (NCBI) database (http://ncbi.nlm.nih.gov, accessed on 1 June 2023) and Genome Assembly Reference (GRCh38) were used as references. The GenBank accession number of the novel VUS is indicated in Table 1.

The first novel variant (c.1019T>C) was detected in a 55-year-old female patient who had a familial history of prostate cancer among first-degree relatives, albeit without any occurrences of breast cancer in the family lineage. The second novel variant (c.2363T>G) was uncovered in a 58-year-old woman who possessed a familial history of breast cancer among first-degree relatives. Notably, only homozygous genotypes (TT and GG) were detected for this specific variant. The third novel variant, designated as c.3192T>C, was identified in a 53-year-old woman with a familial history of breast cancer among first-degree relatives. We were unable to ascertain the segregation of these variants with the phenotype within the patient’s family due to the unavailability of her parents for molecular testing.

### 2.2. In Silico Analysis

In silico prediction of the eventual impact of *BRCA1* exon 11 changes on splice site strength were carried out using the Splice Site Prediction by Neural Network (https://www.fruitfly.org/seq_tools/splice.html), which evaluates the presence and the strength of putative donor and acceptor splice sites [17].

The splicing regulatory elements (SREs) that bind splicing regulatory proteins to regulate local pre-mRNA splicing were analyzed using HExoSplice [18] (http://bioinfo.univ-rouen.fr/HExoSplice/inputs.php) and DeepCLIP [19] (https://deepclip.compbio.sdu.dk/) to predict potential changes in their activity that could enhance or silence splicing in the regions containing the identified VUS.

### 2.3. Cell Cultures 

MCF7 (human breast cancer, ATCC, HTB-22) and HeLa (human cervical carcinoma, ATCC, CCL-2) cell lines were grown in Dulbecco’s modified Eagle’s medium (DMEM, Gibco, Grand Island, NY, USA) with 4500 mg/L Glucose, Pyruvate and L-Glutamine supplemented with 1% penicillin/streptomycin and 10% Fetal Bovine Serum (FBS, Gibco, Grand Island, NY, USA). Cells were incubated at 37 °C in a 5% CO_2_ atmosphere.

### 2.4. Functional Splicing Assays

The pB1 wild-type vector used to test the impact on splicing of the novel *BRCA1* variant was kindly provided by Prof. Diana Baralle [20]. The plasmid contains the sequence from *BRCA1* exon 8 up to the first 89 nucleotides of exon 12. Each of the three *BRCA1* exon 11 variants was generated by gene synthesis (GenScript, Rijswijk, The Netherlands) and sequencing was used to confirm the identity of all minigenes. Wild-type and mutant constructs were transfected into MCF7 and HeLa cells by using Lipofectamine 2000 (Thermo Fisher Scientific, Waltham, MA, USA), according to the manufacturer’s instructions. After two days, cells were harvested and the total RNA was extracted using Trifast reagent (Euroclone, Milan, Italy), according to manufacturers’ protocol.

The purity and concentration of isolated RNA were assessed by spectrophotometric analysis using a NanoDrop One instrument (Thermo Fisher Scientific). One microgram of total RNA with acceptable purity ratios was used for first-strand cDNA synthesis. This reaction utilized M-MLV reverse transcriptase (Thermo Fisher Scientific) and a reverse primer (pCSrev, 5′-GCAACTAGAAGGCACAGTCGAGG-3′) that anneals specifically to the pB1 vector sequence.

Then, cDNA was amplified with Taq DNA Polymerase (New England Biolabs, Ipswich, MA, USA) as described by Tammaro et al. [21], by using the following primers: (1) forward primer [9–10F: 5′-ACTTATTGCAGTGTGGGAGA-3′] annealing with the exon 9/10 junction and a mixture of (2) reverse primer 1, specific for *BRCA1* Exon 11 FL isoform [11FLR: 5’-GGAGTCCGCCTATCATTACATG-3’], hybridizing within exon 11 distal to the 11Q splice site and (3) reverse primer 2 specific for ∆11Q and ∆11 [12R: 5′-GGAGTCCGCCTATCATTACATG-3′], annealing to proximal exon 12 and overlapping the exon 10/12 junction together with the exon 11Q/12 junction [21]. PCR products were visualized using 2.5% (*w*/*v*) agarose gel electrophoresis and images were quantified with ImageJ software (v. 1.53) [22]. Briefly, the optical density peak values were generated with ImageJ software and Excel was used to calculate the percentage splicing index (PSI/ψ) of each variant, where ψ = variant n/((variant n + variant (n + 1) + variant (n + 2)) band intensity. The data are presented as the mean ± standard error (SEM) from three independent experiments. Statistical comparisons were performed by using One-way ANOVA followed by the Tukey test. Probability (*p*) values of <0.05 were considered statistically significant.

To assess the impact of potential splicing factors identified by DeepClip analysis, we conducted transfections in both MCF7 and SKBR3 cells. The vector with the c.2363T>G mutation was transfected at a concentration of 1 μg, along with the control empty vector (pCG, 0.1 µg). Furthermore, cells were transfected with a vector containing either the splicing factors SRSF1 and SRSF10 [23] (0.1 µg) or the splicing factor TIA-1 [24] (0.1 µg). Subsequent analysis of the splicing pattern was performed as described above.

### 2.5. DNA Capillary Electrophoresis

DNA capillary electrophoresis for the analysis of size distribution and relative percentage of PCR products was carried out utilizing the QIAxcel Advanced System (QIAGEN GmbH, Hilden, Germany) according to the manufacturer’s guidelines. The system employed pre-filled cartridges containing a high-resolution gel matrix (QIAxcel DNA High-Resolution Gel Cartridge) to support optimal fragment separation.

For precise sizing of PCR products, a size standard ladder (QX DNA Size Marker, 50 bp–1.5 kb, QIAGEN GmbH) was included in each run alongside the samples. Additionally, an alignment marker (QX Alignment Marker, 15 bp–3 kb, QIAGEN GmbH) was incorporated into every run. The size standard ladder provided reference bands of known sizes for accurate fragment size determination, while the alignment marker ensured comprehensive visualization of migration patterns across a broader size range.

After electrophoresis, data processing was performed using the dedicated software provided with the instrument (QIAxcel ScreenGel Software v.1.2). Subsequently, the generated data were subjected to further analysis using GraphPad Prism 6 to ensure the statistical significance of relative quantitation. The data are presented as the mean from three independent experiments. The statistical significance of the observed differences was assessed relative to the corresponding wild-type isoforms using one-way ANOVA followed by the Tukey test.

## 3. Results and Discussion

### 3.1. Identification of Novel BRCA1 Exon 11 Variants and In Silico Assessment of Pathogenicity

Sequencing of the *BRCA1* gene within a cohort of patients with a family history of cancer revealed the presence of three novel variants (Table 1) previously unreported in studies conducted in Arab countries [16].

In the exploration of *BRCA1* variants, particularly those classified as VUSs, the challenge often lies in deciphering their functional impact on protein behavior. A significant portion of these VUSs comprises rare missense variants, presenting a considerable gap in understanding their implications. In this specific context, functional assays represent pivotal tools to provide a spectrum of evidence, ranging from supportive to robust, for the classification of variants as either pathogenic or benign. Given the multifaceted role of the *BRCA1* protein across various cellular processes, employing a diverse array of assays should be advisable. Consequently, CanVIG-UK guidelines advocate for this approach, highlighting five key functional protein studies, including transcriptional activation (TA) and homology-directed recombination repair (HDR) assays, as essential components for variant interpretation [25].

In parallel, in vitro systems are useful for functional studies and offer a direct observation of mutation effects on cellular processes, enriching our understanding of variant pathogenicity [26,27]. However, in scenarios where experimental characterization proves challenging, computational analyses step in to fill the gap. Our study, for instance, utilized splicing prediction tools to forecast the impact of novel *BRCA1* variants on exon 11 splicing. Though unable to directly probe protein function, these in silico analyses offer valuable insights into potential splicing alterations, thus serving as a crucial preliminary step in variant prioritization.

**Figure 1:**
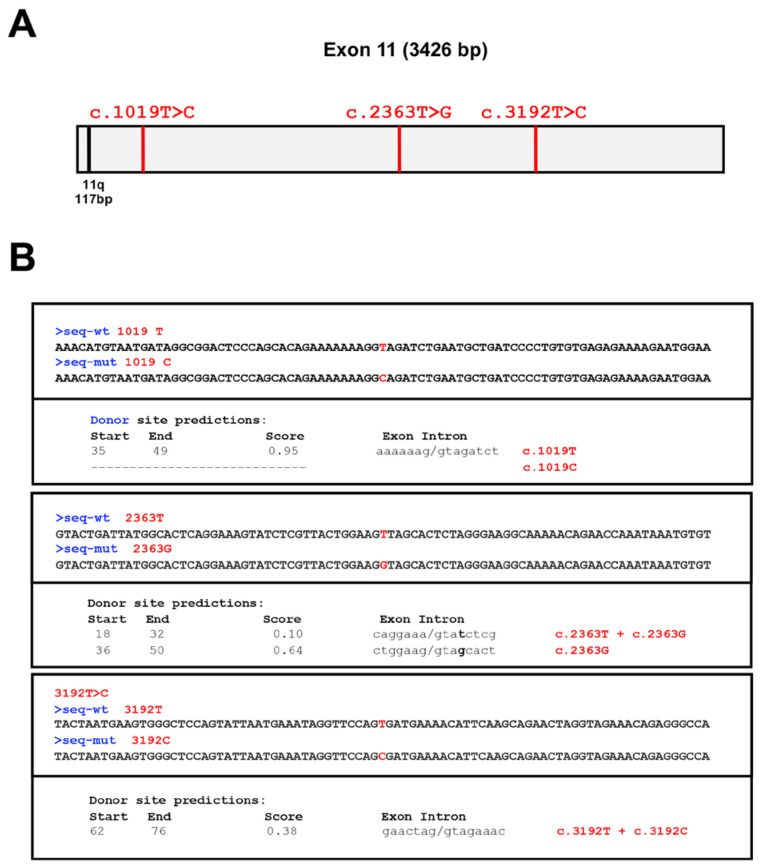
Predictions of VUS’ impact by Splice Site Prediction by the Neural Network (SSPNN) tool. (**A**) Schematic diagram of the human BRCA1 exon 11 (gray box). The positions of the three novel VUS (c.1019T>C, c.2363T>G, and c.3192T>C) within exon 11 are highlighted with red vertical lines. The position of the Δ11q splicing site is highlighted with a black vertical line. (**B**) The numbers (0–1) indicate the range of scores assigned to splice sites by the SSPNN tool. A lower number indicates weak or less conserved splice sites and a higher number indicates strong splice sites. The summary of the analysis (which allele is more likely to skip the exon) is indicated within the main text.

Analysis of splice site strength by Splice Site Prediction by Neural Network analysis predicted that two out of these three variants could alter the splicing process. Specifically, it predicted that the c.1019 T>C variant could impair the quality of the wild-type donor splice site (seq-wt) whilst the c.2363T>G variant could lead to the utilization of a cryptic splice site. In parallel, no changes in splice site strength were predicted for the c.3192T>C variant (Figure 1).

We then used another splicing prediction tool, HExoSplice, to evaluate potential changes in the activity of auxiliary splicing regulatory elements (SREs) that bind splicing factors and regulate local splicing. This analysis suggested that the c.1019 T>C variant (Supplementary Figure S1) could indeed alter the splicing regulatory properties of the region from silencing to enhancing, based on changing the HExoSplice ESRseq scores for hexamer sequences from negative to positive. In contrast, the program predicted that the c.2363T>G transversion could have altered the splicing regulatory properties of the region in which it was located from enhancing to silencing (Supplementary Figure S2). Finally, no substantial changes in the splicing regulatory profiles were detected for the c.3192 T>C variant (Supplementary Figure S3).

Overall, these in silico analyses suggest that the mutation c.2363T>G might cause an alteration in the exon 11 definition compared to the wild-type allele (c.2363T), even without activating cryptic splice sites.

### 3.2. Functional Splicing Assays

**Figure 2:**
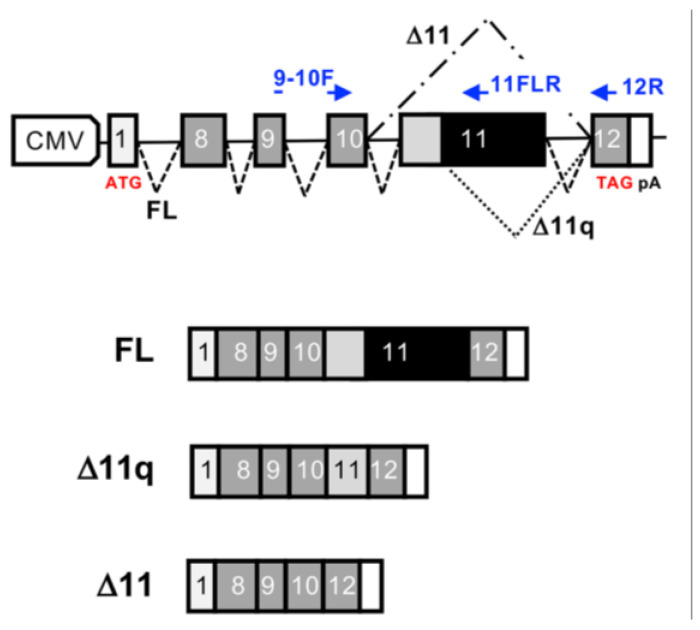
Schematic representation of the minigene splicing construct of *BRCA1* exon 11. In the upper panel, the pB1 wild type (WT) version of the minigene is depicted. The components include CMV, which represents the promoter of the pCDNA3 vector; ATG, indicating the start codon; TAG, representing the stop codon; and pA, indicating the poly-A signal. Exon 1 of the alfa globin gene is denoted as “1”, while BRCA1 exons from 8 to 12 are numbered accordingly. The positions of specific oligos used for detection are shown (in blue). Introns are depicted with black solid lines and alternative splicing of exon 11 is represented by dotted lines (FL, ∆11q, and ∆11). In the lower panel, the three splicing isoforms [full length (FL), ∆11q, and ∆11] are illustrated.

To address these predictions, we employed the pB1 *BRCA1* minigene system that has already been utilized in previous studies [20,28,29] to perform functional investigations (Figure 2, upper panel). In this minigene system, we therefore introduced the three variants (c.1019C, c.2363G, and c.3192C) by mutating the wild-type sequence of *BRCA1* exon 11 (Figure 2, lower panel).

Subsequently, we transfected these novel minigenes into two human breast cancer cell lines (MCF7 and SKBR3). The MCF-7 cell line, derived from human mammary carcinoma, exhibits estrogen receptor (ER) and progesterone receptor (PR) positivity and expresses non-amplified HER-2, making it a representative model for the Luminal A molecular subtype of clinical breast cancer [30,31]. In contrast, the SKBR3 cell line is distinguished by its human epidermal growth factor receptor 2-positive (HER2+) status, characteristic of aggressive breast cancer subtypes [32].

Given these distinctions and considering that splicing factors can behave differently in different cell lines, depending on the local context [33], transfections were carried out in both these cell lines to gain better insight into how specific variants may affect splicing mechanisms across different breast cancer subtypes.

Following minigene transfection, the total RNA from the cells was extracted after a 48-hour incubation period. The processed RNA from the minigene was then reverse-transcribed using a vector-specific primer and amplified using specific primers designed to distinguish between the three splicing isoforms generated by *BRCA1* exon 11 splicing, namely, the full-length (FL), Δ11, and Δ11q isoforms.

**Figure 3:**
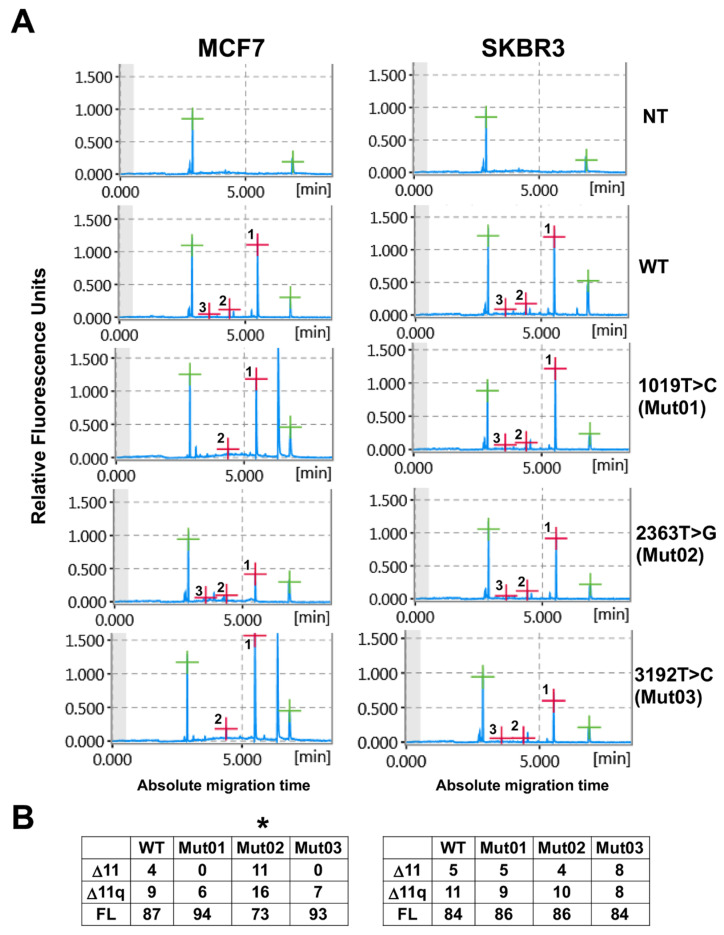
Impact of the novel *BRCA1* novel VUS on exon 11 splicing. (**A**) The detection of different percentages of the main BRCA1 exon 11 splicing isoforms was carried out using capillary electrophoresis. Post-transfection of MCF7 (left panel) and SKBR3 (right panel) cell lines showing the *BRCA1* exon 11 splicing isoforms for not transfected cells (NT), pB1 construct carrying the *BRCA1* exon 11 wild-type, (WT), the 1019T>C (Mut01), the 2363T>G (Mut02), or the 3192T>C (Mut03) substitutions. The positions of the three main splicing isoforms in the electropherograms are denoted with red crosses corresponding to each peak (Full length, FL = 1; Δ11q = 2; and Δ11 = 3). Additionally, the positions of the alignment marker, containing two fragments peaking at 15 bp and 3 kb, are indicated with green crosses. Peak heights represent fluorescence intensity (scale on relative fluorescent units). (**B**) The tables present the relative percentage (%) of each isoform in MCF7 (left panel) and SKBR3 (right panel) calculated against the “total” intensity derived from the sum of the three peak areas, using the QIAxcel ScreenGel Software. Statistically significant splicing variations were observed only for c2363T>G (Mut02) in MCF7 cells (*, *p* Value < 0.05).

PCR products showing different peak sizes were detected in the electropherogram of each sample (Figure 3). Notably, the c.2363T>G mutation was the only variant associated with a significant alteration in the splicing pattern: it displayed a statistically significant decrease in the full-length (FL) isoform, accompanied by a concurrent increase in the levels of Δ11q and Δ11 isoforms (Figure 3, right panel). Interestingly, this effect was not observed in SKBR3 cells (Figure 3, left panel). These findings suggest that the nucleotide alteration c.2363T>G has the potential to modify the exon 11 splicing pattern in a cell-specific manner, presumably due to the fact that the two different cell lines express different sets of splicing factors. This finding underscores the importance of evaluating variants in multiple cell lines whenever feasible, as cell-specific differences in splicing factor expression may contribute to variant-specific effects.

It is worth noting that while our study focused primarily on characterizing the impact of the main BRCA1 exon 11 variants on pre-mRNA splicing, it is possible that other aberrant splicing events exist (i.e., Delta exon 9; Delta exon 10; and Delta exon 9 + 10) such as those outlined in the study where pB1 vector was initially used [20]. However, our study prioritized investigating the known main BRCA1 exon 11 variants as they currently represent the predominant alternative splicing variants with potential biological significance.

Next, to investigate which splicing factors may be implicated in the splicing alteration caused by the c.2363T>G transversion, we utilized the recently developed DeepCLIP tool [19] to predict changes in RNA-protein interactions based on the sequence change. 

Analysis with the pre-trained model for all available splicing factors predicted that the c.2363T>G variant could significantly alter binding to this region of at least four splicing factors: ELAVL1, TIA1 (Figure S4, Upper panel), SRSF1, and SRSF10 (Figure S4, Lower panel). For ELAVL1 and TIA1, the predicted binding ability to the mutated sequence decreased compared to the wild-type (i.e., decreased mutated/wild-type score ratio). Conversely, for SRSF1 and SRSF10, the predicted binding ability increased compared to the mutated sequence (i.e., increased mutated/wild-type score ratio). Interestingly, these splicing factors have been shown to regulate both exon inclusion and skipping. Specifically, ELAVL1 has been reported to cooperatively control exon inclusion in transcripts like CD44, also relevant in breast cancer [34]. Likewise, TIA1 was reported to promote exon inclusion like for SMN2 exon 7 and its absence could alter this splicing program [35]. The role of SRSF10 as either a splicing activator or a repressor is unclear; however, some studies have shown that it can bind to specific exons and promote their skipping. For example, SRSF10 could mediate the skipping of exon 6 in the CDC25A gene [36,37].

Finally, the splicing factor SRSF1 has been shown to regulate both exon inclusion and skipping, suggesting that it can act as either a splicing activator or repressor [38]. Specifically, SRSF1 has been reported to promote the skipping of certain exons, including exon 11 of the RON receptor, exon BIN1, MKNK2, and CASC4 [39]. Moreover, SRSF1 is upregulated in human breast tumors and its overexpression can promote the transformation of mammary cells [39]. Given its known role in regulating alternative splicing events, including exon skipping, the prediction that SRSF1 binding may be altered by the c.2363T>G variant supports the hypothesis that this change could lead to aberrant splicing associated with this mutation. The dual functionality of SRSF1 as both an activator and repressor of splicing makes it a relevant candidate that could be involved in the splicing alteration predicted to occur with the c.2363T>G transversion identified here. Therefore, this computational prediction supports the hypothesis that one or more of these splicing factors, with known roles in regulating alternative splicing, may be implicated in the splicing alteration associated with the c.2363T>G variant identified here.

To validate the potential impact of these splicing factors, we conducted experiments involving transfections of MCF7 and SKBR3 cells with vectors overexpressing SRSF1 and SRSF10 or TIA1. Despite the predictions suggesting that alterations in splicing patterns could be due to these factors, no significant changes in the alternative splicing of the c.2363T>G mutant were observed in our experimental assays. It should be noted, however, that splicing is a highly combinatorial process. Therefore, lack of action by varying single splicing factors may not necessarily recapitulate the splicing effects observed in cellular environments. Indeed, in our specific case, the observation that the splicing affecting variants has an effect in one cell line tested but not in the other suggests that the variation in single factors is unlikely to explain this behavior and further work will have to be conducted to address this issue satisfactorily.

## 4. Conclusions

The landscape of *BRCA1* exon 11 mutations is expanding, particularly in regions like Africa, where limited resources and restricted access to genetic testing still prevail [40,41,42,43,44,45,46]. In our specific breast cancer context, it is well-established that germ-line mutations in the *BRCA1* gene significantly elevate the risk of its development. While screening for SNPs within exon 11 is crucial for diagnosis and prognosis, establishing functional assays to assess the functional impact of these changes at the protein level poses challenges. Nonetheless, understanding the influence of SNPs on protein–protein interactions is a complex issue, both experimentally and computationally [13,47,48]. Experimental approaches like direct mutagenesis studies using CRISPR/CAS9 can be employed to ascertain their effects on protein function but they tend to be time-consuming and not always feasible.

In this study, the mutations c.1019T>C and c.2363T>G were of particular interest due to their potential effects on both protein function and pre-mRNA splicing. Computational methods suggested harmful effects at the splicing level, with one variant being validated using a minigene-based assay. These findings emphasize the importance of considering the impact of variants on pre-mRNA splicing when examining BRCA1 mutations.

**Figure 4:**
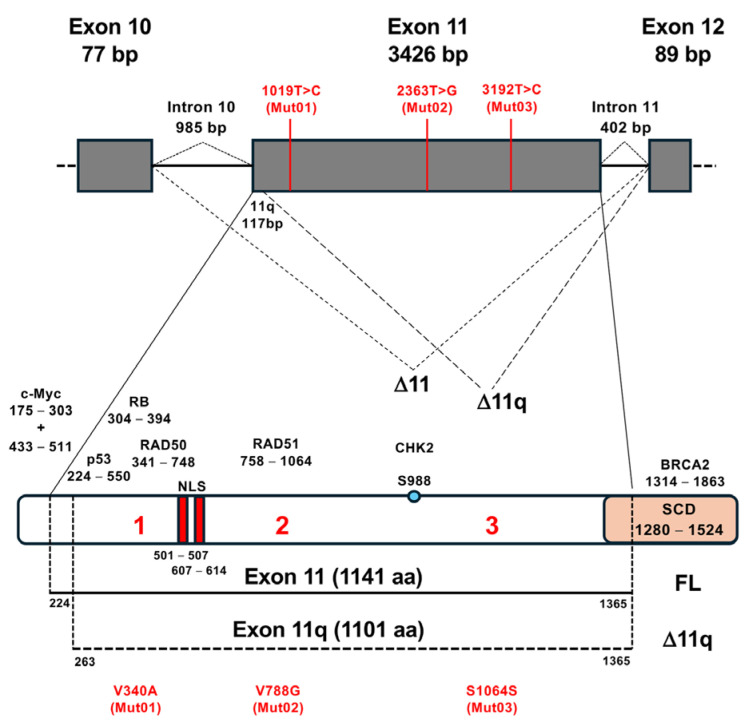
Schematic diagram of the genomic structure and binding partners at the protein level of the human BRCA1 exon 11. Upper panel shows the genomic region of *BRCA1* spanning from exon 10 to exon 12. Exons 10, 11, and 12 are represented by gray boxes, while introns are indicated by black solid lines, with their sizes specified in base pairs (bp). Dotted lines illustrate alternative splicing of exon 11, showcasing the three splicing isoforms FL, Δ11q, and Δ11. The positions of the three novel VUS (1019T>C, 2363T>G, and 3192T>C) within exon 11 are highlighted in red. Lower panel shows the schematic diagram of *BRCA1* full-length exon 11 (FL) and the protein versions of Δ11q (only the protein region spanning exon 11 is displayed) are presented. Functional domains such as the Nuclear Localization Signal (NLS) and Serine Containing Domain/SQ Cluster Domain (SCD/SQCD) are delineated. Interactors of exon 11, involving Rb, p53, c-Myc, Rad50, Rad51, and BRCA2 (illustrating the sites where the BRCA1 protein interacts with these proteins), are also depicted, along with the phosphorylation site crucial for DNA damage signaling and the kinase responsible for its modification, CHK2. The positions of the three novel VUS within exon 11 at the protein level are shown with red numbers (1019T>C = V340A = 1; 2363T>G = V788G = 2; and 3192T>C = S1064S = 3). The c.2363T>G mutation causes an amino acid change (V788G) within the region critical for interaction with the RAD51 factor. The c.1019T>C mutation causes an amino acid change (V340A) within the region critical for interaction with RB and RAD50 factors.

The observed decrease in the percentage of full-length BRCA1 exon 11 underscores the potential impact of alterations at both pre-mRNA and protein levels, potentially affecting the protein’s levels and functions. In fact, of particular significance is the exploration of potential pathogenic variants within exon 11 of the *BRCA1* gene, the largest exon containing two NLS [3] and regions interacting with crucial transcription factors (such as MYC, Rb, and p53) and proteins involved in DNA repair (RAD50 and RAD51) (Figure 4) [2,49]. In this context, the c.2363T>G mutation is expected to cause an amino acid change (V788G) within the domain critical for interaction with the RAD51 factor (Figure 4). 

These considerations lead us to hypothesize that the pathogenicity of VUS c.2363T>G might be the result of a combination of alterations at both the splicing and protein level, ultimately leading to a reduction in full-length BRCA1 exon 11 and changes in the structural conformation of the BRCA1 protein.

Consequently, a combination of alterations of BRCA1 exon 11 has the potential to synergistically disrupt interactions critical for cell cycle regulation and DNA repair mechanisms, thereby compromising genome stability. This disruption in the RNA analysis and protein sequence may thus elevate the lifetime risk of developing breast and ovarian cancer. More specifically, the absence of a fully functional NLS in the Δ11q protein isoform could lead to its retention in the cytoplasm, potentially triggering cell proliferation [4].

Another interesting point is the difference in the splicing pattern associated with the c.2363T>G mutation in two distinct human breast cancer cell lines, MCF7 and SKBR3, each characterized by different ER and HER2 profiles. Interestingly, these findings suggest that the impact of a single point mutation on splicing and consequently on protein function may vary according to the specific cellular context and underscore the potential role of the heterogeneity of breast cancer types and stages in regulating alternative splicing [50,51,52].

The significant role of splicing factors in breast cancer has been emphasized by their capacity to regulate splice site selection and modulate the alternative splicing pattern of genes associated with cancer. Furthermore, certain splicing factors can have oncogenic properties, suggesting that the impact of a single-point mutation on splicing and protein function may be influenced by the expression levels of these factors [53].

Moreover, in recent years, it has been documented that mutations targeting the binding sites of splicing factors on pre-mRNA can disrupt the proper function of the spliceosome, leading to aberrant alternative splicing of genes associated with cancer [54,55]. These elements add a further layer of complexity to the mechanisms underlying the development and progression of breast cancer.

The discrepancy between the predictions of potential splicing factors and experimental results highlights the complexity of splicing regulation. Splicing is governed by a network of splicing factors and regulatory elements, whose interplay is influenced by various factors such as post-transcriptional modifications, protein–protein interactions, and cellular microenvironments [56,57,58]. It is conceivable that the overexpression of individual splicing factors may not suffice to elicit significant changes in splicing patterns, particularly in the presence of endogenous regulatory mechanisms within different cellular contexts. The integration of in silico predictions and experimental investigations underscores the need for further studies to unravel the intricate dynamics underlying splicing alterations associated with the c.2363T>G variant. In addition, as this mutation is novel and has not been previously described, further research involving a larger cohort of women carrying this mutation will be essential to quantify the associated cancer risk.

Overall, our research highlights the importance of evaluating the potential consequences of VUS not only at the protein level but also in terms of splicing. Due to the intricate nature of pre-mRNA splicing decisions, functional assays are indispensable to definitively address the question of potential pathogenicity. Consequently, splice variant analysis should represent a valuable addition in diagnostic genetic laboratories to benefit the clinical management of patients with these variations.

## Figures and Tables

**Table 1:** Novel genetic variants identified in exon 11 of the *BRCA1* gene in Libyan breast cancer patients. The table shows the type of variation (missense/synonymous); the resulting amino acid change; and the GenBank identification number as described in a recent preprint [16].

Variant	Variation Type	AA Change	GenBank ID
c.1019T>C	Missense	V340A	MW716257 (C)
c.2363T>G	Missense	V788G	MW716260 (G)
c.3192T>C	Synonymous	S1064S	MW716258 (T)

## Data Availability

Data is contained within the article or Supplementary Materials.

## Supplementary Materials

The following supporting information can be downloaded at: https://www.mdpi.com/article/10.3390/cells13100824/s1, Figure S1: In silico analysis of the splicing regulatory change in the BrcA1 exon 11 caused by the c.1019T>C transition; Figure S2: In silico analysis of the splicing regulatory change in the BrcA1 exon 11 caused by the c.2363T>G transversions; Figure S3: In silico analysis of the splicing regulatory change in the BrcA1 exon 11 caused by the c.3192T>C transition; Figure S4: Predicted binding strength of splicing factors ELAVL1, TIA1, SRSF1, and SRSF10 for c.2363T>G transversion using DeepCLIP.
