# Supplementary material for: Assessing the Impact of Novel BRCA1 Exon 11 Variants on Pre-mRNA Splicing
Source: Cells. 2024 May 11;13(10):824. doi: 10.3390/cells13100824 (PMC11119505; doi:10.3390/cells13100824)
Supplement: Supplementary file 1 [file cells-13-00824-s001.zip › cells-2874604-supplementary.pdf]

cells

MDPI

>seq-wt 1019 T
AAACATGTAATGATAGGCGGACTCCCAGCACAGAAAAAAAGGTAGATCTGAATGCTGATCCCCTGTGTGAGAGAAAAGAATGGAA
>seq-mut 1019 C
AAACATGTAATGATAGGCGGACTCCCAGCACAGAAAAAAAGGCAGATCTGAATGCTGATCCCCTGTGTGAGAGAAAAGAATGGAA

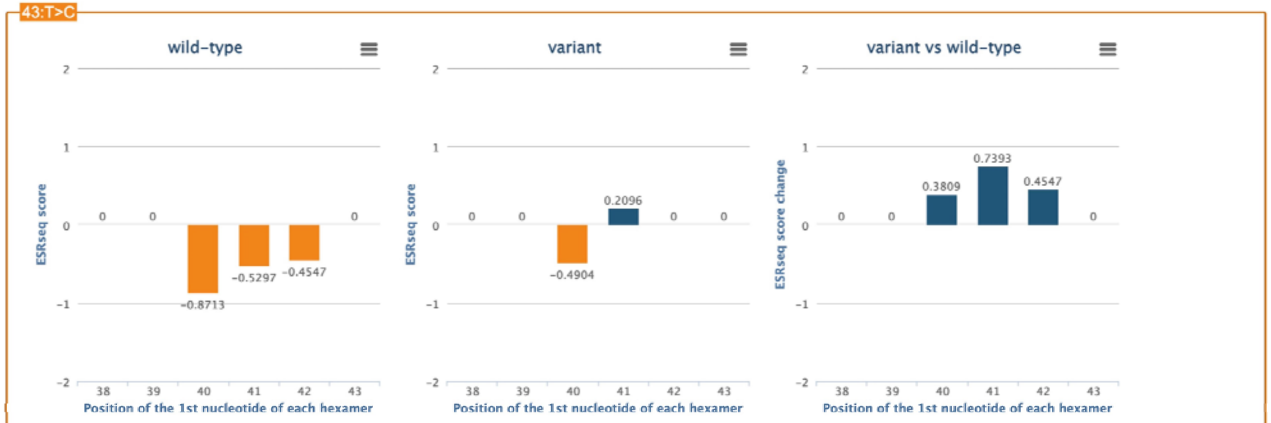

Positions	Hexamer seq wild-type	ESRseq score wild-type	Hexamer assignment wild-type	Hexamer seq variant	ESRseq score variant	Hexamer assignment variant	ESRseq score change (variant vs wild-type)
38-43	AAAGGT	0	N	AAAGGC	0	N	0
39-44	AAGGTA	0	N	AAGGCA	0	N	0
40-45	AGGTAG	-0.8713	S	AGGCAG	-0.4904	S	+0.3809
41-46	GGTAGA	-0.5297	S	GGCAGA	+0.2096	E	+0.7393
42-47	GTAGAT	-0.4547	S	GCAGAT	0	N	+0.4547
43-48	TAGATC	0	N	CAGATC	0	N	0
Total	AAAGGTAGATC	-1.8557	-	AAAGGCAGATC	-0.2808	-	+1.5749

Figure S1. In silico analysis of the splicing regulatory change in the BrcA1 exon 11 caused by the c.1019T>C transition.
HExoSplice2 analysis was performed on an 85 nucleotide sequence surrounding the c.1019T>C variant at position 42. The analysis shows the transition results in a change from negative to positive ESRseq scores for hexamer sequences. This suggests the c.1019T>C variant may promote exon inclusion by switching the splicing regulatory factors binding nearby from repressors to activators of BRCA1 exon 11 splicing.

```
>seq-wt  2363T
GTACTGATTATGGCACTCAGGAAAGTATCTCGTTACTGGAAGTTAGCACTCTAGGGAAGGCAAAAACAGAACCAAATAAATGTGT
>seq-mut  2363G
GTACTGATTATGGCACTCAGGAAAGTATCTCGTTACTGGAAGGTAGCACTCTAGGGAAGGCAAAAACAGAACCAAATAAATGTGT
```

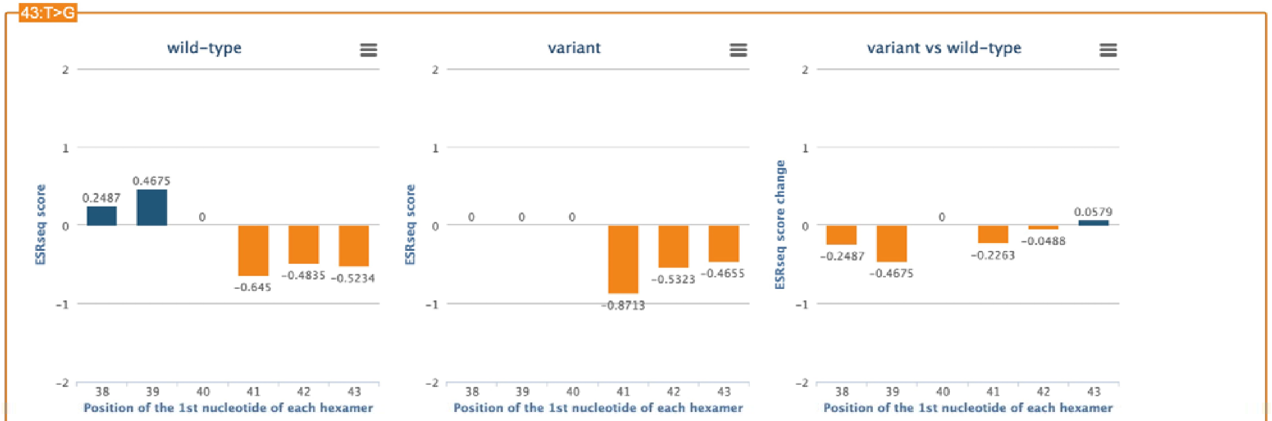

Positions	Hexamer seq wild-type	ESRseq score wild-type	Hexamer assignment wild-type	Hexamer seq variant	ESRseq score variant	Hexamer assignment variant	ESRseq score change (variant vs wild-type)
38-43	GGAAGT	+0.2487	E	GGAAGG	0	N	-0.2487
39-44	GAAGTT	+0.4675	E	GAAGGT	0	N	-0.4675
40-45	AAGTTA	0	N	AAGGTA	0	N	0
41-46	AGTTAG	-0.6450	S	AGGTAG	-0.8713	S	-0.2263
42-47	GTTAGC	-0.4835	S	GGTAGC	-0.5323	S	-0.0488
43-48	TTAGCA	-0.5234	S	GTAGCA	-0.4655	S	+0.0579
Total	GGAAGTTAGCA	-0.9357	-	GGAAGGTAGCA	-1.8691	-	-0.9334

Figure S2. In silico analysis of the splicing regulatory change in the BrcA1 exon 11 caused by the c.2363T>G transversions.
HExoSplice2 analysis was performed on an 85 nucleotide sequence surrounding the c.2363T>G variant at position 42. The analysis shows the transversion results in lower ESRseq scores for hexamer sequences, indicating increased silencing properties of splicing regulatory factors binding nearby. This suggests the c.2363T>G variant may promote exon skipping.

>seq-wt 3192T
TACTAATGAAGTGGGCTCCAGTATTAATGAAATAGGTTCCAGTGATGAAAACATTCAAGCAGAACTAGGTAGAAACAGAGGGCCA
>seq-mut 3192C
TACTAATGAAGTGGGCTCCAGTATTAATGAAATAGGTTCCAGCGATGAAAACATTCAAGCAGAACTAGGTAGAAACAGAGGGCCA

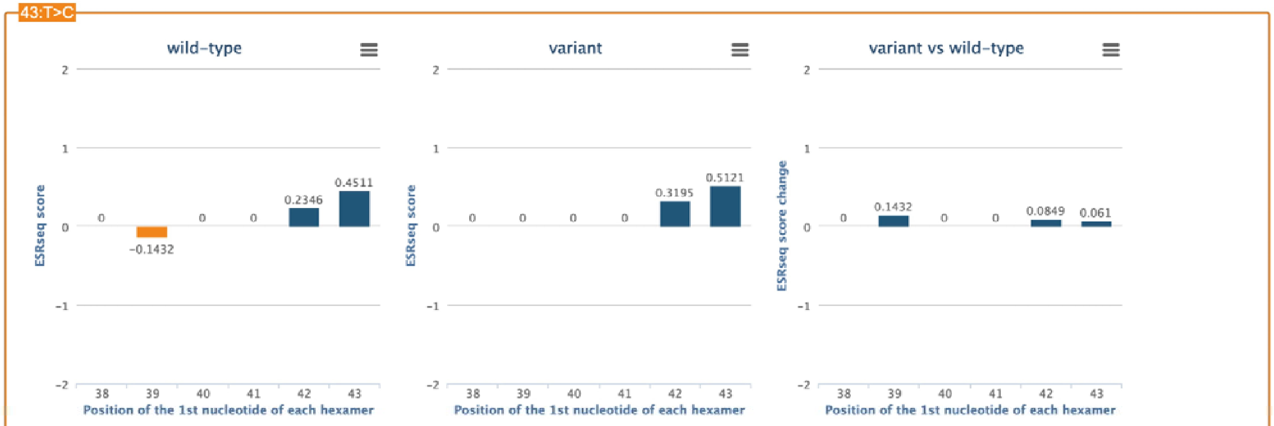

Positions	Hexamer seq wild-type	ESRseq score wild-type	Hexamer assignment wild-type	Hexamer seq variant	ESRseq score variant	Hexamer assignment variant	ESRseq score change (variant vs wild-type)
38-43	TCCAGT	0	N	TCCAGC	0	N	0
39-44	CCAGTG	-0.1432	S	CCAGCG	0	N	+0.1432
40-45	CAGTGA	0	N	CAGCGA	0	N	0
41-46	AGTGAT	0	N	AGCGAT	0	N	0
42-47	GTGATG	+0.2346	E	GCGATG	+0.3195	E	+0.0849
43-48	TGATGA	+0.4511	E	CGATGA	+0.5121	E	+0.0610
Total	TCCAGTGATGA	+0.5425	-	TCCAGCGATGA	+0.8316	-	+0.2891

Figure S3. In silico analysis of the splicing regulatory change in the BrcA1 exon 11 caused by the c.3192T>C transition.
HExoSplice2 analysis was performed on 85 nucleotide sequence surrounding the c.3192T>C variant located at position 42. The analysis shows the transition has minimal effect on predicted binding sites for exonic splicing regulatory factors. No dramatic changes in ESRseq scores for hexamer sequences were observed, with the overall positive trend being maintained. This suggests the c.3192T>C variant is unlikely to significantly alter recruitment of splicing factors that modulate inclusion of BRCA1 exon 11.

Protein	Model_id	id	Sequence	Score	Ratio mut/wt
ELAVL1	RNCMPT00112_RNCMPT	seq-wt 2363T	CAGGAAAGTATCTCGTTACTGGAAGTTAGCACTCTAGGGAAGGCAAAAACA	0.000293629	**0.02330**
ELAVL1	RNCMPT00112_RNCMPT	seq-mut 2363G	CAGGAAAGTATCTCGTTACTGGAAGGTAGCACTCTAGGGAAGGCAAAAACA	6.84293E-06	
TIA1	RNCMPT00165_RNCMPT	seq-wt 2363T	CAGGAAAGTATCTCGTTACTGGAAGTTAGCACTCTAGGGAAGGCAAAAACA	0.007331813	**0.09948**
TIA1	RNCMPT00165_RNCMPT	seq-mut 2363G	CAGGAAAGTATCTCGTTACTGGAAGGTAGCACTCTAGGGAAGGCAAAAACA	0.000729374	

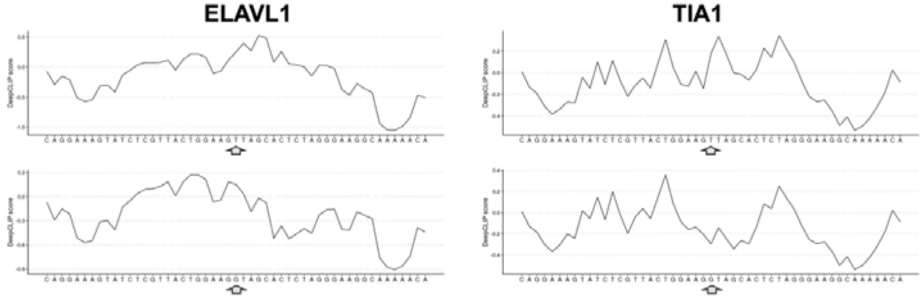

Protein	Model_id	id	Sequence	Score	Ratio mut/wt
SRSF1	RNCMPT00108_RNCMPT	seq-wt 2363T	CAGGAAAGTATCTCGTTACTGGAAGTTAGCACTCTAGGGAAGGCAAAAACA	0.076844051	5.57634
SRSF1	RNCMPT00108_RNCMPT	seq-mut 2363G	CAGGAAAGTATCTCGTTACTGGAAGGTAGCACTCTAGGGAAGGCAAAAACA	0.428508878	
SRSF10	RNCMPT00088_RNCMPT	seq-wt 2363T	CAGGAAAGTATCTCGTTACTGGAAGTTAGCACTCTAGGGAAGGCAAAAACA	0.001381437	2.67794
SRSF10	RNCMPT00088_RNCMPT	seq-mut 2363G	CAGGAAAGTATCTCGTTACTGGAAGGTAGCACTCTAGGGAAGGCAAAAACA	0.003699406	

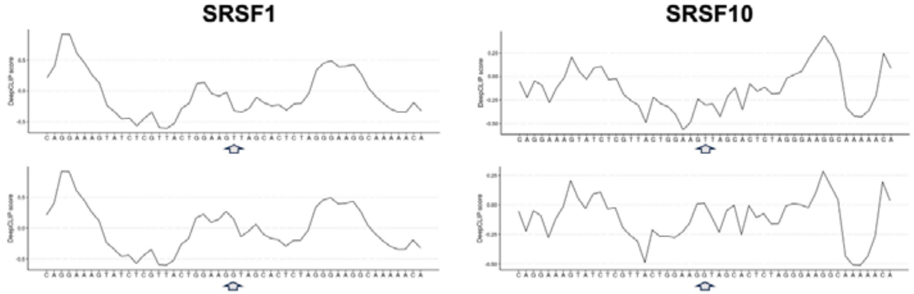

Figure S4. Predicted binding strength of splicing factors ELAVL1, TIA1, SRSF1, and SRSF10 for c.2363T>G transversion using DeepCLIP.

Analysis by DeepCLIP (https://deepclip-web.compbio.sdu.dk/) using the pretrained models of the indicated splicing factors and 85-nt sequences (-42/+42 around the variation). The profiles for the different factors (ELAVL1, TIA1, SRSF1, and SRSF10) predict the binding strength of each of them for the c.2363T>G transversion (indicated by a gray arrow) versus the relative seq-wt sequence.
